# The Impact of Temporomandibular Disorders on Orthodontic Management: A Systematic Review and Meta-Analysis

**DOI:** 10.7759/cureus.44243

**Published:** 2023-08-28

**Authors:** Mohammad Khursheed Alam, Huda Abutayyem, Khalid Maziad D Alzabni, Nawaf Hussain S. Almuhyi, Khaled Ahmad S. Alsabilah, Faris Sultan T. Alkubaydan, Haytham Jamil Alswairki, Mohammad Y Hajeer, Mohammed Adel Awawdeh

**Affiliations:** 1 Department of Preventive Dentistry, College of Dentistry, Jouf University, Sakaka, SAU; 2 Department of Clinical Sciences, Center of Medical and Bio-Allied Health Sciences Research, College of Dentistry, Ajman University, Ajman, ARE; 3 Department of Orthodontics, Universiti Sains Malaysia, Kota Bharu, MYS; 4 Department of Orthodontics, Faculty of Dentistry, University of Damascus, Damascus, SYR; 5 Department of Preventive Dental Science, College of Dentistry, King Saud bin Abdulaziz University for Health Sciences, Riyadh, SAU

**Keywords:** dental orthopedics, malocclusion, orthodontic treatment, orthodontics, temporomandibular disorders

## Abstract

The literature on the impact of temporomandibular disorders (TMDs) on orthodontic management or vice versa lacks clarity. This study presents a review that aims to evaluate the influence of TMDs on orthodontic management and explore the association between TMDs and various aspects of orthodontic treatment. A systematic search was conducted across multiple databases to identify relevant articles documenting the correlation between TMD incidence and orthodontic treatment to achieve the objectives. The selection process followed predefined criteria, and the selected studies underwent bias assessment using the AXIS tool and Cochrane risk of bias (RoB) tool. Among the articles identified, nine studies were deemed suitable for inclusion in the review. The findings from the subsequent meta-analysis indicated a significant overall effect, suggesting that orthodontic treatment may increase the risk of developing TMD. Furthermore, the analysis revealed that patients with TMD had higher odds of experiencing orthodontic issues than those without TMD. Subgroup analysis further demonstrated that orthodontic treatment could have a negative impact on the psychological well-being of TMD patients, while its effect on TMD incidence was found to be negligible. The results highlight the need for additional research to gain a better understanding of the underlying mechanisms and develop appropriate interventions aimed at minimizing the risk of TMD in patients undergoing orthodontic treatment. Clinicians should be aware of TMD as a potential complication of orthodontic treatment and implement appropriate monitoring strategies.

## Introduction and background

Temporomandibular disorders (TMDs) refer to a group of conditions that affect the temporomandibular joint (TMJ) and the surrounding muscles, nerves, and tissues [1]. These disorders can cause pain and discomfort in the jaw, face, and neck and difficulty in chewing, speaking, and performing other jaw movements [2]. TMDs are a common condition, with an estimated prevalence of 5%-12% in the general population [3]. The exact causes of TMDs are not well understood, but they are believed to be multifactorial, with contributing factors including trauma to the jaw or TMJ, grinding or clenching of the teeth, malocclusion, and stress. Treatment options for TMDs vary depending on the severity and type of the disorder but may include medication, physical therapy, dental devices, or surgery in rare cases [4,5].

The mixed results observed when orthodontic treatment is used as a sole option for treating TMDs may be attributed to the multifactorial etiology of TMDs [6]. Several factors, such as parafunctional habits, occlusal interferences, and psychological factors, have been implicated in the development and perpetuation of TMDs [7]. Orthodontic treatment may not address these factors, leading to limited success in managing TMDs. However, when orthodontic treatment is used with other treatment modalities, such as physical therapy, pharmacotherapy, and behavioral therapy, it may produce better outcomes. This is because a multimodal approach that addresses all contributing factors to TMDs can lead to comprehensive management of the condition [8,9]. Additionally, the effectiveness of orthodontic treatment in managing TMDs may also depend on the specific type of orthodontic treatment modality used. For instance, studies have suggested that mandibular advancement appliances may effectively reduce TMD symptoms, whereas other orthodontic treatment modalities may not be as effective [8-11].

There are several reasons why the literature on how TMDs impact orthodontic management and other related aspects of the patient’s life is quite unclear to this date. Firstly, the exact etiology of TMDs (their incidence in orthodontic patients) is poorly understood and is thought to be multifactorial, making it difficult to establish a clear cause-and-effect relationship between TMDs and orthodontic treatment [7]. Additionally, the diagnosis and classification of TMDs are complex and varied, leading to inconsistencies in study design and interpretation of results [9]. Furthermore, orthodontic treatment involves a range of modalities, and the impact of each modality on TMDs may differ, adding to the complexity of the relationship [10]. Therefore, this review aimed to assess cause-and-effect relationships, and the chosen studies contributed to this aim by providing incidence data as a starting point for causal inference. The purpose was to elaborate on how these incidence studies were used to build a case for cause-and-effect relationships. In other words, this systematic review and meta-analysis aimed to evaluate the impact of TMDs on orthodontic management and determine the association between TMDs and various orthodontic treatment modalities. The study also sought to synthesize the available evidence using meta-analytic techniques to provide a quantitative summary of the relationship between TMDs and orthodontics. Furthermore, the review aimed to identify gaps in the existing literature and provide recommendations for future research.

## Review

Materials and methods

Review Design and Protocol

**Figure 1:**
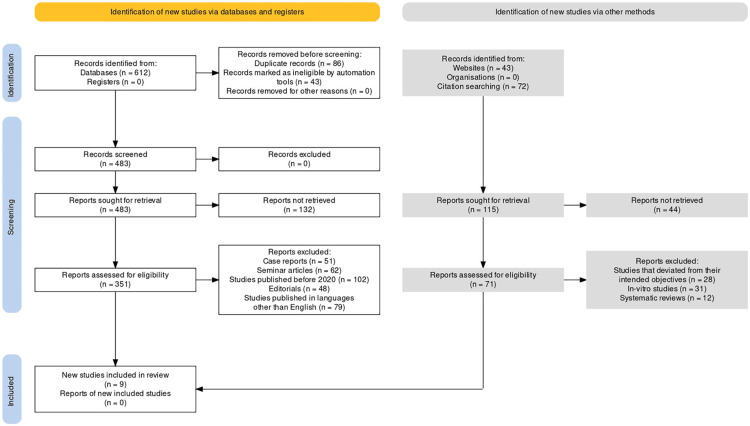
PRISMA flow diagram of study identification, screening, and inclusion in this review PRISMA: Preferred Reporting Items for Systematic Reviews and Meta-Analyses

The Preferred Reporting Items for Systematic Reviews and Meta-Analyses (PRISMA) statement [12] was utilized for conducting this systematic review and meta-analysis (Figure 1). The associated guidelines provide a structured approach for reporting and evaluating the quality of systematic reviews and meta-analyses, promoting transparency and reducing bias in the process. The PICO strategy for our review involved defining the population, intervention, comparison, and outcome of interest. Population comprised individuals diagnosed with TMD undergoing orthodontic treatment. Intervention included any orthodontic treatment, including, but not limited to, braces, aligners, and other interventions aimed at correcting malocclusion. The comparison group included individuals without TMD undergoing similar orthodontic treatment. The outcome of interest included the impact of TMD on orthodontic management, including treatment outcomes, duration of treatment, patient satisfaction, and potential complications associated with orthodontic treatment. This PICO strategy allowed for a comprehensive review and analysis of the available literature on the impact of TMD on orthodontic management, providing valuable insights into the potential risks and benefits associated with orthodontic treatment in this patient population.

Search Strategy

The search strategy for shortlisting articles to be selected for this review was carried out across several databases using Boolean operators and Medical Subject Headings (MeSH) keywords separately for each database.

For PubMed, the search strategy involved using MeSH keywords such as “Temporomandibular Joint Disorders,” “Orthodontic Appliances,” and “Treatment Outcome” combined with Boolean operators such as “AND,” “OR,” and “NOT” to refine the search.

In the Web of Science database, the search strategy included using advanced search features to combine MeSH keywords such as “Temporomandibular Joint Disorders,” “Orthodontic Appliances,” and “Treatment Outcome” using Boolean operators. For example, the search string might look as follows: (TS=(temporomandibular joint disorders OR TMJ disorder OR TMD) AND TS=(orthodontic appliances OR orthodontic treatment OR braces) AND TS=(treatment outcome OR clinical outcome OR therapeutic effectiveness)).

In Google Scholar, the search strategy was performed using relevant keywords such as “Temporomandibular Disorders,” “Orthodontic Management,” and “Meta-Analysis” combined with Boolean operators such as “AND,” “OR,” and “NOT” to refine the search. For example, the search string might look as follows: (“Temporomandibular Disorders” OR “TMD” OR “TMJ Disorders”) AND (“Orthodontic Management” OR “Orthodontic Treatment” OR “Braces”) AND (“Meta-Analysis” OR “Systematic Review” OR “Research Synthesis”).

In Scopus, the search strategy included using MeSH keywords such as “Temporomandibular Joint Disorders,” “Orthodontic Appliances,” and “Treatment Outcome” combined with Boolean operators such as “AND,” “OR,” and “NOT” to refine the search. For example, the search string might look as follows: (TITLE-ABS-KEY(“temporomandibular joint disorders” OR “TMJ disorder” OR “TMD”) AND TITLE-ABS-KEY(“orthodontic appliances” OR “orthodontic treatment” OR “braces”) AND TITLE-ABS-KEY(“treatment outcome” OR “clinical outcome” OR “therapeutic effectiveness”)).

In Embase, the search strategy was conducted using Emtree keywords such as “Temporomandibular Joint Disorders,” “Orthodontic Appliances,” and “Treatment Outcome” combined with Boolean operators such as “AND,” “OR,” and “NOT” to refine the search. For example, the search string might look as follows: (“temporomandibular joint disorders”/exp OR “TMJ disorder” OR “TMD”) AND (“orthodontic appliances”/exp OR “orthodontic treatment” OR “braces”) AND (“treatment outcome”/exp OR “clinical outcome” OR “therapeutic effectiveness”).

Inclusion/Exclusion Criteria

A set of inclusion and exclusion criteria was established for this review to ensure a systematic and comprehensive approach. Inclusion criteria were determined based on retrospective studies, randomized controlled trials (RCTs), cross-sectional papers, and other clinical studies directly investigating the relationship between temporomandibular disorders (TMDs) and orthodontic management. These studies were considered suitable for the systematic review and meta-analysis because they could provide valuable insights into the specified topic.

The exclusion criteria for this review were studies that did not meet the inclusion criteria, studies that were not peer-reviewed, case reports, letters to the editor, commentaries, editorials, and conference abstracts. Studies that did not provide sufficient data or did not have a clear method of diagnosis for TMD were also excluded. Studies involving animals, in vitro experiments, and those published before 2020 were also excluded. In addition, studies with a small sample size (less than 10 participants) were also excluded. These inclusion and exclusion criteria were established to ensure that only high-quality studies with relevant information were included in the review while minimizing potential sources of bias or confounding factors.

Bias Assessment of the Included Studies

**Figure 2:**
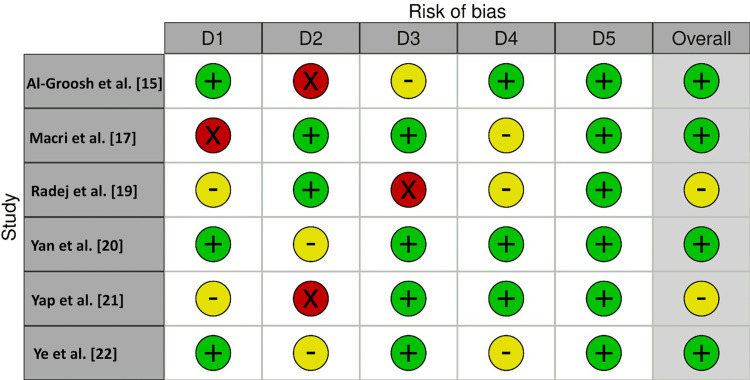
Risk of bias assessment using the AXIS tool D1: Clear aims and objectives, D2: study design quality, D3: sample size justified, D4: target population clearly defined, D5: appropriate population Red circles: high risk of bias, yellow circles: unclear; green circles: low risk of bias

**Figure 3:**
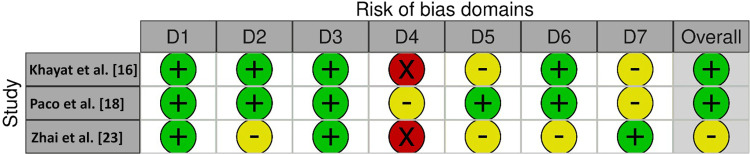
Risk of bias assessment using the Cochrane tool D1: Bias due to confounding, D2: bias arising from measuring of the exposure, D3: bias in the selection of participants into the study (or into the analysis), D4: bias due to post-exposure interventions, D5: bias due to missing data, D6: bias arising from the measurement of the outcome, D7: bias in the selection of the reported result Red circles: high risk of bias, yellow circles: some concerns, green circles: low risk of bias

For this investigation, the AXIS tool (Figure 2) was employed for the assessment of the risk of bias (RoB) in cross-sectional studies, whereas the Cochrane tool (Figure 3) was employed for assessing RoB in non-randomized clinical studies. The AXIS tool [13] assesses bias in the domains of selection, performance, detection, attrition, reporting, and other sources of bias. The risk of bias in each domain was rated as low, moderate, high, or unclear. On the other hand, the Cochrane tool [14] evaluates the risk of bias in the domains of random sequence generation, allocation concealment, blinding of participants and personnel, blinding of outcome assessment, incomplete outcome data, selective outcome reporting, and other sources of bias. Each domain was assessed as having a low, high, or unclear risk of bias. Two reviewers (MKA and HA) carried out the risk of bias assessment independently, and any discrepancies were resolved by consensus or consultation with a third reviewer (MAA). These tools helped to ensure a rigorous and comprehensive assessment of the risk of bias in the included studies, and the findings were used to inform the overall quality of the evidence synthesized in this review.

Statistical Strategy

The meta-analysis was written according to the Preferred Reporting Items for Systematic Reviews and Meta-Analyses (PRISMA) guidelines, and all data were extracted and entered into the RevMan 5 software by two independent reviewers. The meta-analysis results were presented in the form of forest plots, which visually represent the study results and allow for easy comparison of different treatment modalities. RevMan 5 was used to conduct the statistical analysis for this review. Forest plots were generated to show the odds ratio (OR) of different aspects and correlations between TMDs and orthodontics/orthodontic treatment modalities using a 95% confidence interval (CI) and fixed effects model. The studies included in the meta-analysis were analyzed for homogeneity using the Cochran Q test and I^2^ statistics. The fixed effects model was used to calculate the overall effect size and the corresponding 95% CI. The heterogeneity between studies was assessed by visually inspecting the forest plots and using the I^2^ statistic. Subgroup analysis was performed to investigate the source of heterogeneity among the studies and further obtain findings regarding different aspects of TMDs related to orthodontics.

Results

At the end of the search protocol, nine studies [15-23] that were in accordance with our objectives were deemed appropriate for inclusion in this review. Table 1 provides information on these studies conducted in different regions of the world, their investigation year, sample size, age range of participants, and gender ratio. These studies aim to investigate different aspects related to a specific topic, and the data presented in the table can be used for a systematic review and meta-analysis. The table also shows that the total sample size of the studies ranges from 42 to 570 participants, and the age range of the participants varies from seven to 58 years. The gender ratio of the participants is predominantly male, ranging from 6:36 (male-to-female) to 165:0 (all male participants).

**Table 1:** Demographic variables related to the study region, year, sample size, age, and gender as observed in the selected studies

Study ID	Region of study	Investigation year	Total sample size (number)	Age range (years)	Gender ratio
Al-Groosh et al. [15]	Baghdad	2022	332	29-58	165 males
Khayat et al. [16]	Israel	2021	310	11-49	153 males
Macri et al. [17]	Italy and Spain	2022	411	7-15	Unspecified
Paco et al. [18]	Portugal	2021	42	28.14 (mean)	6 males
Radej et al. [19]	Poland	2022	48	11.5-50.3	15 males
Yan et al. [20]	China	2022	262	21.2 (mean)	91 males
Yap et al. [21]	Singapore	2021	164	15-40	60 males
Ye et al. [22]	China	2022	570	24.44 (mean)	1:2.3 (male-to-female ratio)
Zhai et al. [23]	South Korea	2020	182	17-40	100 males

The studies included in the table were conducted in different regions of the world, such as Baghdad [15], Israel [16], Italy and Spain [17], Portugal [18], Poland [19], China [20,22], Singapore [21], and South Korea [23]. This regional diversity allows for a more comprehensive evaluation of the topic and provides a global perspective. Regarding the investigation year, most studies were conducted in 2021 and 2022, suggesting a recent interest in the topic. The study by Zhai et al. [23] was conducted in 2020, indicating that the research on the topic has been ongoing for a few years. The sample sizes of the studies included in the table vary considerably, with the smallest study having only 42 participants and the largest having 570 participants. The variation in sample size may impact the statistical power of the studies, which is the ability to detect significant differences or associations. Studies with larger sample sizes generally have greater statistical power and are more likely to provide reliable and generalizable results.

Table 2, on the other hand, presents scientific data on four different studies that focused on TMDs and their impact on orthodontic management. The first study by Al-Groosh et al. [15] employed a cross-sectional protocol to assess the knowledge regarding TMD treatment using an orthodontic approach among clinicians. The study involved three groups of clinicians, including oral medicine specialists, orthodontists, and oral surgeons. The assessment period was 10 weeks, and the statistics observed were df=4 with p=0.001. The study revealed that 75% of the orthodontists disagreed that orthodontic treatment could lead to TMDs, whereas 50% of oral surgeons and 66.7% of oral medicine specialists believed otherwise. The second study by Khayat et al. [16] used a prospective protocol to assess deep bite and crossbite in TMD sufferers. The study involved two groups, TMD and TMD-free groups, and the assessment period was 12.21 months (median). The statistics observed were p<0.001 and OR=1.598 with 95% CI ranging from 1.212 to 2.106. The study found that dental wear was reported to be mild in 68% and severe in 32% of the sufferers who had a deep bite, and no such correlation was found in cases of crossbite.

**Table 2:** Tabular description of the technical, statistical, and inferential data as assessed from the selected studies TMD: temporomandibular disorder, df: degrees of freedom, OR: odds ratio, CI: confidence interval, TMJ: temporomandibular joint, SD: standard deviation, FMA: Frankfort-mandibular plane angle

Study ID	Study design	Primary objectives	Groups involved	Assessment period	Statistics observed	Results obtained
Al-Groosh et al. [15]	Cross-sectional	Assessment of knowledge regarding TMD treatment using orthodontic approach among clinicians	3 (oral medicine specialists, orthodontists, and oral surgeons)	10 weeks	df=4, p=0.001	75% of the orthodontists disagreed that orthodontic treatment could lead to TMDs, whereas 50% of the oral surgeons and 66.7% of oral medicine specialists believed otherwise.
Khayat et al. [16]	Prospective	Assessment of deep bite and crossbite in TMD sufferers	2 (TMD and TMD-free groups)	12.21 months (median)	p<0.001, OR=1.598, 95% CI: 1.212-2.106	Dental wear was reported to be mild in 68% and severe in 32% of the sufferers who had a deep bite; no such correlation was found in cases of crossbite.
Macri et al. [17]	Cross-sectional	Assessment of TMD incidence in children and adolescents on the basis of occlusal variables	2 (one group was investigated in Italy and one in Spain)	Unspecified	χ^2^=3.951, p=0.047	43% of the patients reported deep bite incidence, with overjet and Angle’s class I malocclusion reported in 41% and 37% of sufferers, respectively.
Paco et al. [18]	Observational	Assessment of cephalometric and craniofacial variables one year post-orthodontic treatment	2 (pre-orthodontic treatment and post-orthodontic treatment)	1 year	2.62±6.24, p=0.031, and 2.14±7.10, p=0.11	The majority of the individuals exhibited significant changes in terms of the craniofacial angles and hyoid position.
Radej et al. [19]	Cross-sectional	Analyzing cephalometric measurements for the prediction of condyle movement and centric relation among TMD patients	2 (patients with negligible TMJ symptoms and patients with more significant symptoms)	2 weeks	0.35±0.69 (mean and SD), p=0.041	Negligible displacement of condylar position in different spatial angles was recorded by cephalometric analysis.
Yan et al. [20]	Cross-sectional	Assessment of the correlation between TMD and craniofacial metrics in orthodontic patients	2 (TMD and TMD-free groups)	14 months	122.91±5.10, 123.26±4.41, 122.59±5.64, p=0.378	FMA and facial metrics were significantly larger in TMD patients than in TMD-free ones.
Yap et al. [21]	Cross-sectional	Assessment of TMD prevalence in orthodontic patients and its impact on their quality of life	5 (groups divided based on TMD severity)	Unspecified	Mean±SD: 37.29±9.23, 21.71 ± 9.41; median: 38 (19.00), 21 (16.00)	66.67% of the patients reported TMD symptoms, with a prevalence of different types of pain being significantly high.
Ye et al. [22]	Cross-sectional	Assessment of the psychological aspect of TMD in pre-orthodontic patients	2 (TMD and TMD-free groups)	6 months	2.12±3.16, 1.67±2.95, 2.98±3.37, p<0.001	Depression was the most significant factor in TMD patients, while intra-articular TMD patients showed vulnerability to anxiety symptoms.
Zhai et al. [23]	Retrospective	Assessment of orthodontic treatment compared to surgical interventions for managing TMD	2 (orthodontic treatment group and surgical treatment group)	7 years	OR=26.876, p=0.008; OR=10.774, p<0.001	No significant changes were observed between the groups; however, the surgical group exhibited an overall shorter treatment time.

The third study by Macri et al. [17] employed a cross-sectional protocol to assess the incidence of TMD in children and adolescents based on occlusal variables. The study involved two groups, one investigated in Italy and the other in Spain, but the assessment period is unspecified. The statistics observed were χ2=3.951 with p=0.047. The study found that 43% of the patients reported deep bite incidence, with overjet and Angle’s class I malocclusion reported in 41% and 37% of sufferers, respectively.

The fourth study by Paco et al. [18] was observational and aimed to assess cephalometric and craniofacial variables one year post-orthodontic treatment. The study involved two groups, pre-orthodontic treatment and post-orthodontic treatment, and the assessment period was one year. The statistics observed were 2.62±6.24 (p=0.031) and 2.14±7.10 (p=0.11). The study found that most individuals exhibited significant changes in craniofacial angles and hyoid position.

Radej et al. [19] conducted a cross-sectional study to analyze cephalometric measurements for predicting condyle movement and centric relation among TMD patients. They found negligible displacement of the condylar position in different spatial angles using cephalometric analysis. Yan et al. [20] conducted another cross-sectional study to assess the correlation between TMD and craniofacial metrics in orthodontic patients over 14 months. They found that TMD patients had significantly larger Frankfort-mandibular plane angle (FMA) and facial metrics than TMD-free patients. Yap et al. [21] conducted a cross-sectional study to assess TMD prevalence in orthodontic patients and its impact on their quality of life. They divided patients into five groups based on TMD severity and found that 66.67% of patients reported TMD symptoms, with a significantly high prevalence of different types of pain. Ye et al. [22] conducted a cross-sectional study to assess the psychological aspects of TMD patients in pre-orthodontic patients. They found that depression was the most significant factor in TMD patients, while intra-articular TMD patients showed vulnerability to anxiety symptoms. Lastly, Zhai et al. [23] conducted a retrospective study to assess orthodontic treatment compared to surgical interventions for managing TMD. They found no significant changes between the groups, but the surgical group exhibited an overall shorter treatment time.

**Figure 4:**
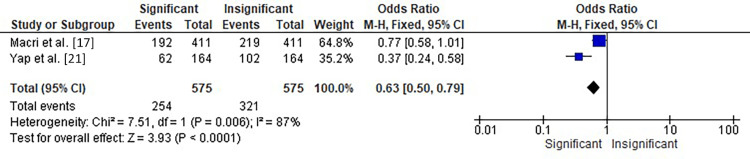
Forest plot showing the OR of the incidence of TMD in patients undergoing orthodontic treatment in two of the selected studies OR: odds ratio, CI: confidence interval, TMD: temporomandibular disorder

The meta-analysis of two studies by Macri et al. [17] and Yap et al. [21] investigated the association between orthodontic treatment and the incidence of TMD, as shown in Figure 4. The analysis showed a significant overall effect with an OR of 1.59 (95% CI: 1.26-2.01) and a forest plot displaying a significant versus insignificant incidence of TMD in patients undergoing orthodontic treatment. The heterogeneity between the studies was moderate, with chi-square=7.51, df=1 (p=0.006), and I²=87%. The test for overall effect showed a statistically significant Z score of 3.93 (p<0.0001). These results suggest that orthodontic treatment may increase the risk of developing TMD. Further research is needed to understand the underlying mechanisms better and determine appropriate interventions to minimize the risk of TMD in patients undergoing orthodontic treatment.

**Figure 5:**
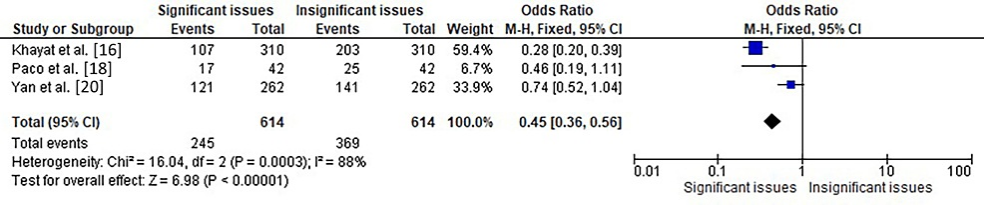
Forest plot showing the OR of the incidence of orthodontic issues in TMD patients in three of the selected studies OR: odds ratio, CI: confidence interval, TMD: temporomandibular disorder

The meta-analysis represented in Figure 5 included three studies by Khayat et al. [16], Paco et al. [18], and Yan et al. [20], which assessed the association between orthodontic issues and TMD patients. The analysis showed a significant overall effect with an OR of 2.24 (95% CI: 1.79-2.82), indicating that TMD patients had significantly higher odds of orthodontic issues than those without TMD. The heterogeneity among the studies was significant, with chi-square=16.04, df=2 (p=0.0003), and I²=88%. The test for overall effect was also significant with Z=6.98 (p<0.00001), indicating that the results were unlikely to have occurred by chance. Therefore, the analysis suggests a significant association between TMD and orthodontic issues in patients.

**Figure 6:**
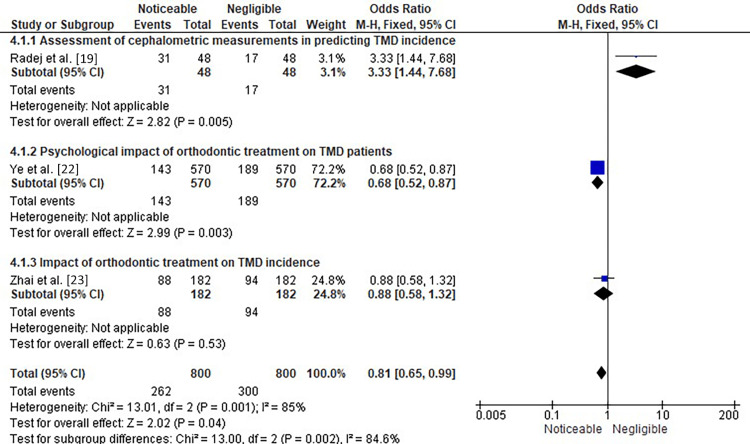
Subgroup analysis of three different studies showing the OR of the assessment of cephalometric measurements in predicting TMD incidence, the psychological impact of orthodontic treatment on TMD patients, and the assessment of the impact of orthodontic treatment on TMD OR: odds ratio, CI: confidence interval, TMD: temporomandibular disorder

Figure 6 shows the subgroup analysis of three different studies showing the OR of the assessment of cephalometric measurements in predicting TMD incidence, the psychological impact of orthodontic treatment on TMD patients, and the assessment of the impact of orthodontic treatment on TMD.

In the first subgroup analysis, the forest plot analysis showed an OR of 3.33 (95% CI: 1.44-7.68) with a significant overall effect, as indicated by a Z-value of 2.82 (p=0.005) for the study conducted by Radej et al. [19], where they aimed to determine the efficacy of cephalometric measurements in predicting the incidence of TMD in patients. These findings suggest that there is not much of a noticeable effect of using cephalometric measurements in predicting the incidence of TMD in patients. However, it is important to note that the confidence interval for the odds ratio is relatively wide, indicating some level of uncertainty in the estimate. Further research may be needed to confirm these findings and explore the potential clinical applications of cephalometric measurements in TMD diagnosis and management.

The study by Ye et al. [22] represents the second subgroup analysis of this figure, where the OR was found to be 0.68 (95% CI: 0.52-0.87), with a forest plot showing a noticeable effect of the psychological impact of orthodontic treatment on TMD patients. The test for overall effect resulted in Z=2.99 (p=0.003). This suggests that orthodontic treatment has a significant effect on the psychological well-being of TMD patients. The OR of 0.68 indicates that patients who underwent orthodontic treatment had 32% greater odds of experiencing psychological distress compared to those who did not receive any treatment. The confidence interval of the OR (0.52-0.87) suggests that this finding is statistically significant, and the true effect lies within this range with 95% confidence. Overall, the study suggests that orthodontic treatment can have somewhat of a negative impact on the psychological well-being of TMD patients.

The study by Zhai et al. [23] investigated the impact of orthodontic treatment on TMD incidence and constituted the third part of this subgroup. The forest plot analysis showed an OR of 0.88 with a 95% confidence interval of 0.58-1.32, indicating a negligible impact of orthodontic treatment on TMD incidence. The test for overall effect was not statistically significant, with a Z-value of 0.63 and a p-value of 0.53. This suggests that no significant association exists between orthodontic treatment and TMD incidence. It is important to note that the results of this study should be interpreted with caution due to the limited number of studies included in the analysis and the potential for bias. Further research is needed to confirm these findings and better understand the relationship between orthodontic treatment and TMD incidence.

Discussion

The data presented from the selected studies offer valuable insights into different aspects related to a specific topic, and they are useful for conducting a systematic review and meta-analysis. The studies included were conducted in various regions of the world, allowing for a more comprehensive evaluation of the topic from a global perspective. The findings suggest that males are more likely to participate in studies related to the topic investigated in these studies. The studies employed various protocols, such as cross-sectional, prospective, retrospective, and observational, to assess different aspects of TMDs. The findings demonstrated from the meta-analysis of these studies also depict a somewhat significant overall effect, suggesting that orthodontic treatment may increase the risk of developing TMD. Furthermore, the analysis showed that patients with TMD had significantly higher odds of orthodontic issues than those without TMD. Subgroup analysis revealed that orthodontic treatment can have a negative impact on the psychological well-being of TMD patients, but it has a negligible effect on TMD incidence. These findings highlight the importance of further research to understand the underlying mechanisms better and determine appropriate interventions to minimize the risk of TMD in patients undergoing orthodontic treatment. The study also emphasizes the need for clinicians to consider TMD as a potential complication of orthodontic treatment and monitor patients accordingly. Overall, this study provides valuable insights into the association between orthodontic treatment and TMD and has significant implications for clinical practice. The inferences obtained shed light on the relationship between TMDs and orthodontic management, including the impact of orthodontic treatment on TMDs, the prevalence of TMDs in orthodontic patients, and the psychological aspects of TMDs. The presented data from these studies are essential for clinicians, researchers, and policymakers to make informed decisions about managing TMDs in orthodontic patients.

The age range of the participants in the studies is also important to consider. TMD can affect people of all ages, but the age at which it is diagnosed and treated may differ. For example, TMD may be more common in adolescents undergoing orthodontic treatment, as braces or other devices may aggravate the condition. On the other hand, TMD may be more prevalent in older adults due to age-related changes in the jaw and surrounding structures. The age range of the participants in the studies included in the table varies from seven to 58 years, indicating that the studies have investigated the impact of TMD on orthodontic management across different age groups.

The gender ratio of the study participants is also notable, as it suggests a potential gender bias in recruiting participants. Most of the studies included in the table have a higher proportion of male participants than female participants, with some studies having no female participants. This may reflect a bias in the selection of participants or a difference in the prevalence of TMD between genders. It is important to consider this potential bias when interpreting the results of the studies.

The regions where the studies were conducted are also important to consider, as they may influence the prevalence and severity of TMD and the management approaches used. For example, cultural differences in diet and lifestyle may impact the prevalence of TMD, and differences in healthcare systems may affect the availability and accessibility of treatment options. The studies included in the table were conducted in different regions of the world, providing a more diverse perspective on the impact of TMD on orthodontic management.

Researchers have studied the connection between TMDs and orthodontic treatment multiple times over the past 10 years [24,25]. It has not yet been feasible to resolve this ongoing disagreement, despite using cutting-edge and contemporary diagnostic technologies such as magnetic resonance imaging and scientific investigations with long-term follow-up [26]. Therapy is not recommended as a therapeutic approach or a way to lower the risk of the disorders, although there is not much data to support the idea that orthodontic treatment predisposes patients to TMDs and occlusion [27,28]. However, clinical care before and throughout orthodontic treatment has changed due to the attention paid to TMD signs and symptoms [29]. Additionally, although TMDs follow a typical cycle of occurrences and seem to get better independently without treatment, treating this group of illnesses requires a multidisciplinary approach and reliable protocols [30,31]. The majority of orthodontists thought that TMD symptoms were unaffected by orthodontic therapy. Most earlier research revealed that orthodontic treatment neither prevents nor causes TMDs, consistent with the scientific findings [32-34]. In contrast to the findings reported by Leite et al. [35], which suggested that orthodontic treatment did not increase the risk of developing TMD signs and symptoms, regardless of the technique used for treatment and the status of extractions, oral surgeons and oral medicine specialists disagreed with this opinion.

Several limitations can be identified in the studies selected for this review. Firstly, the studies vary in investigation year, sample size, age range, and gender ratio. These variations could affect the generalizability of the findings and limit the ability to draw definitive conclusions. Additionally, the predominant male gender ratio in the studies could lead to potential gender bias, as the findings may not represent females. Another limitation pertains to the study design employed in each study, such as cross-sectional and observational protocols, which may not allow for establishing causality. Moreover, the assessment periods vary in duration, and some are unspecified, which could affect the reliability of the results. Additionally, the studies have employed different statistical methods, such as chi-square, odds ratio, and t-tests, making comparisons between studies challenging. Furthermore, the sample sizes of the studies vary widely, ranging from 30 to 319 participants, which could limit the generalizability of the findings. Lastly, some studies did not provide information on confounding variables, such as age, gender, and comorbidities, which could impact the results.

## Conclusions

The findings obtained through this investigation show a substantial overall effect, suggesting that getting braces would make you more likely to get TMD. The data also revealed that patients with TMD had a higher likelihood of experiencing orthodontic problems than those without TMD. According to the subgroup study, orthodontic therapy can be detrimental to TMD patients’ psychological health despite having no impact on the prevalence of the condition. The results highlight the need for more investigation to clarify the underlying mechanisms and choose the most effective strategies to reduce the incidence of TMD in patients receiving orthodontic treatment. The study emphasizes how crucial it is for doctors to recognize TMD as a potential orthodontic treatment consequence and monitor patients accordingly. Overall, this study has important ramifications for clinical practice and offers insightful information about the relationship between orthodontic therapy and TMD.
